# Gut microbiota metabolism disturbance is associated with postoperative atrial fibrillation after coronary artery bypass grafting

**DOI:** 10.1038/s44325-024-00003-z

**Published:** 2024-06-03

**Authors:** Yuhua Liu, Zhiyong Du, Yingyuan Lu, Ying Ma, Yunxiao Yang, Florian Osmanaj, Yifan Zhang, Xiaoyu Guo, Yanwen Qin, Xiubin Yang, Kun Hua

**Affiliations:** 1 Beijing Institute of Heart Lung and Blood Vessel Disease, Beijing Anzhen Hospital, Capital Medical University, Beijing, China; 2 State Key Laboratory of Natural and Biomimetic Drugs, School of Pharmaceutical Sciences, Peking University, Beijing, China; 3 State Key Laboratory for Quality Ensurance and Sustainable Use of Dao-di Herbs, National Resource Center for Chinese Materia Medica, China Academy of Chinese Medical Sciences, Beijing, China

**Keywords:** Cardiovascular diseases, Endocrine system and metabolic diseases

## Abstract

Postoperative atrial fibrillation (POAF) is a common complication after coronary artery bypass grafting (CABG) surgery. Gut microbiota and its metabolites have been implicated in the development of AF. However, whether the gut–host metabolic interaction contributes to POAF is still unknown. This study aimed to investigate the POAF-associated gut microbiota metabolism biomarkers and related risk model. The POAF (*N* = 30) patients and non-POAF (*N* = 60) patients from the discovery cohort exhibited significantly different microbiome and metabolome profiles. The differentiated features were mainly implicated in the bile acids (BAs) and short-chain fatty acids metabolism, inflammation, and oxidative stress. Random forest analysis identified the combination of five secondary BAs showed a powerful performance on predicting POAF in the discovery cohort, highlighting significant values of area under the curve (AUC = 0.954) and correct classification rate (CCR, 93.3%). In addition, the five secondary BAs-based risk model also exhibited good performance in differentiating the POAF (*N* = 114) and non-POAF individuals (*N* = 253) in an independent validation cohort (AUC = 0.872; CCR = 90.4%). This work revealed perturbed microbial and metabolic traits in POAF, providing potential avenues for the prediction and prevention of POAF after CABG.

## Introduction

Postoperative atrial fibrillation (POAF) is a common complication after cardiac surgery, and it also occurs after non-cardiac thoracic surgery^1^. In general, atrial fibrillation (AF) events postoperatively developed between days 2 and 4 after cardiac operation^2^. The incidence of POAF varies from 20% to 50% depending on the type of cardiac surgery^3^. Various pathological mechanisms have been reported to be associated with POAF, such as vulnerable atrial substrate, activation of the autonomic nervous system, inflammation, and oxidative stress^2^. Previous studies have reported that POAF is associated with increased short-term and long-term morbidity and mortality, as well as an elevated risk of subsequent AF, stroke, and overall surgery costs^4,5^. Many preventive treatments have been suggested in the clinical practice, but the prevalence of POAF remains substantial^5,6^. Therefore, the precise and crucial mechanisms of POAF still need to be further explored in order to find new therapeutic targets.

Metabolomics, aims to characterize diverse classes of the small molecule chemical entities involved in metabolism, has been widely used to identify biomarkers in the diagnosis and prognosis of disease and discover active drivers of biological and pathological processes^7,8^. In the past decade, the state-of-the-art metabolomic analyses performed on a variety of bio-sample types have improved our ability to understand the pathologies of various cardiovascular diseases^9,10^. More recently, by performing targeted metabolite profiles on the pericardial fluid and serum samples from patients undergoing isolated coronary artery bypass grafting (CABG) with and without POAF, we identified several oxidative stress-related metabolites were significantly altered between POAF and non-POAF patients^11^.

The gut microbiota is vital to maintaining human health^12^. Human microbiome represents a metabolically bioactive community, and its composition and function play an important role in regulating host metabolism homeostasis and pathogenesis^12^. The gut microbiome produces a variety of metabolites, such as short-chain fatty acids (SCFAs), indoles, and secondary Bas^13–15^. Gut-heart axis is a novel emerging concept based on the interaction between the gut microbiota and the heart that occurs via biologically active metabolites of bacterial origin, which are resorbed in the intestine and distributed via the circulation^16,17^. Furthermore, accumulating evidence revealed that the alterations in the gut microbiota-derived metabolites could influence the pathophysiological processes of cardiovascular disease, such as coronary heart diseases, arrhythmia, and heart failure^18–21^.

**Fig. 1:**
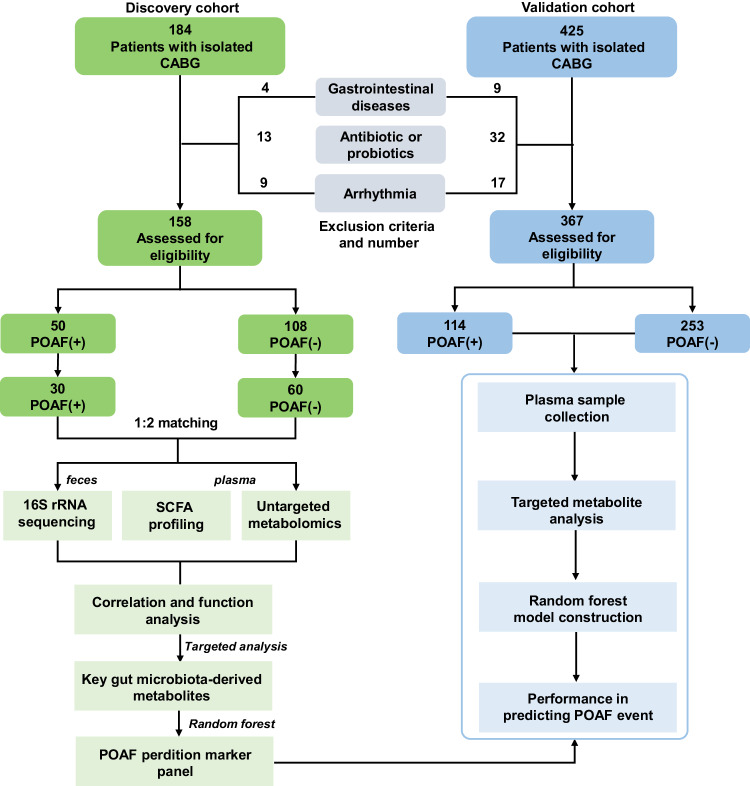
Flow chart of the study design. CABG coronary artery bypass grafting, POAF post-operative atrial fibrillation, SCFA short-chain fatty acid.

Although several small-scale clinical studies demonstrated that the perturbed gut microbiota composition or altered microbial metabolites were associated with the onset and progression of atrial fibrillation^22,23^, the changes in the microbiome and its derived metabolites in POAF are poorly understood. Herein, we presented our study to first and comprehensively explore the fecal microbiome and plasma metabolome of patients undergoing isolated CABG with and without POAF. We sought to explore the POAF-associated alterations in the gut bacteria and metabolites and provide novel insights into pathogenesis for POAF. We also aimed to identify early and precise diagnosis markers for POAF and provide novel treatment avenues for POAF prevention. The overall experimental design for this study is shown in Fig. 1.

## Results

### Baseline characteristics of the discovery cohort

A total of 158 patients (male, 54.8%) undergoing isolated CABG were enrolled in the discovery cohort (Fig. 1). Fifty patients developed POAF in post-operation 2–4 days. The demographics and clinical characteristics of patients with and without POAF are presented in Supplementary Table 1. Notably, compared to patients without POAF, patients who developed POAF had significantly longer hospital length of stay (*P* < 0.05). Although there was no statistical significance in the gender between the two groups, patients who developed POAF were more likely to be male (*P* = 0.055). Due to the incomplete consistency of baseline clinical features, we employed propensity score matching (PSM) algorithm to select individuals for further omics analysis. After 1:2 PSM, a total of 30 patients with POAF and 60 patients without POAF were included for plasma metabolome and fecal microbiome analyses. No statistical differences were observed between the two matched groups (Supplementary Table 1).

### Patients with POAF are characterized by disordered gut microbiota diversity

**Fig. 2:**
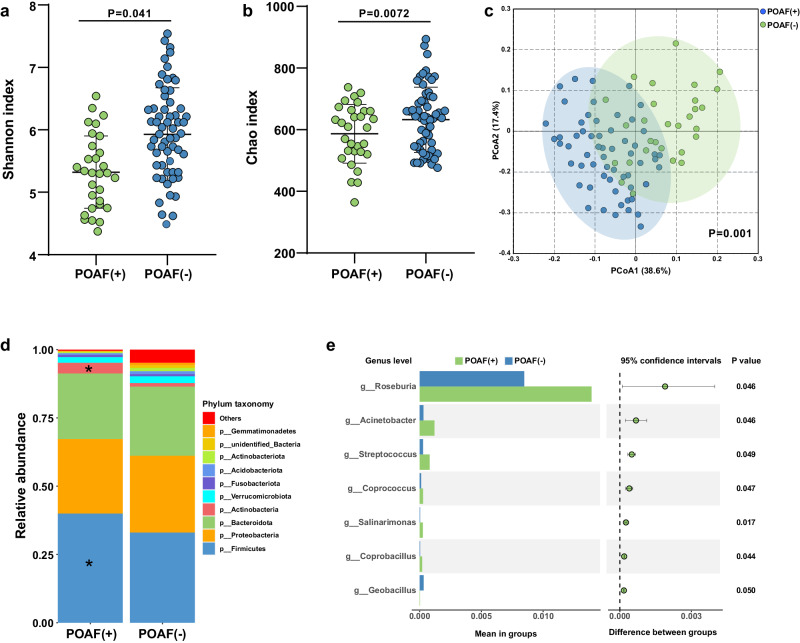
Fecal microbiota profiles of subjects with and without POAF. **a**, **b** Differences in alpha-diversity of gut microbiota between POAF and non-POAF patients, including Shannon index (diversity) and Chao 1 index (microbial community richness). **c** The β diversity of POAF and non-POAF patients based on the PCoA analysis. **d** Differentiated microbiome composition at phylum level in POAF and non-POAF fecal samples. **e** Relative abundance comparison at genus level between the POAF and non-POAF patients, the box plot indicates the mean proportions, the bar plot indicates the proportion differences between two groups.

The gut microbiota profiling of 30 POAF patients and matched 60 no-POAF patients were performed using 16 S rRNA sequencing. To evaluate the microbial diversity differences between POAF and non-POAF groups, alpha and beta diversity analyses were performed. As shown in Fig. 2a, b, a significant difference in Shannon diversity and Chao index was observed between POAF and non-POAF groups (*P* values < 0.05), which demonstrated a significantly lower diversity and community richness in the feces of patients with POAF than in those without POAF. Furthermore, PCoA score plot demonstrated a significant difference in β-diversity between the POAF and non-POAF groups (*P* = 0.001), suggesting that the microbial composition in patients with POAF was significantly shifted regarding OTUs (Fig. 2c). Examination of the microbiome at the phylum level demonstrated that Actinobacteria and Firmicutes were significantly enriched in patients with POAF compared with non-POAF controls (Fig. 2d). At genus level, *Roseburia*, Acinetobacter, Streptococcus, Coprococcus, Salinarimonas, and Coprobacillus were significantly enriched in POAF (Fig. 2e).

### The metabolome map of patients with POAF is characterized by a variety of alterations in bile acid metabolism

**Fig. 3:**
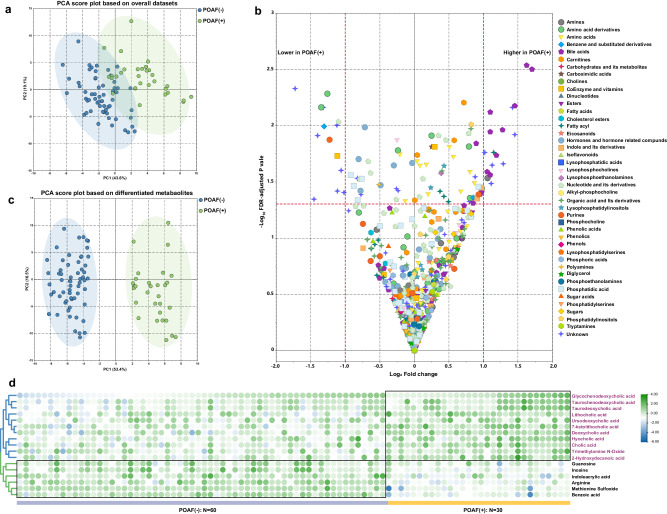
Patients with POAF exhibit profound alterations in gut microbial metabolites. **a**, **b** PCA and PLS-DA score plots based on the datasets from the untargeted metabolomic analysis of plasma samples of patients with and without POAF. **c** The VIP and p(corr) combination plot. The whole set of metabolites is ranked according to their VIP values, *p* (corr) values. VIP > 1.5 or absolute *p* (corr) values > 0.4 represents a significant importance of the metabolic variables in differentiating POAF and non-POAF. **d** Heatmap of the differentiated metabolites that contributed to the discrimination of POAF from non-POAF. p(corr) modeled covariation p-scaled correlation, FA fatty acids, CAR acyl carnitines, LPA lysophosphatidic acid, LPC lysophosphocholine, LPE lysophosphoethanolamines.

Given the interplay between the gut microbiome and host metabolism, then, we performed untargeted metabolomics on plasma samples from POAF and non-POAF groups. After data preprocessing, a total of 621 circulating metabolites and 125 unknown ion variables were detected in the untargeted metabolomic profiling. The identified metabolites mainly include carnitines, fatty acids, BAs, purines, amino acid and their derivatives, organic acids, carbohydrate and their derivatives, nucleotide and their derivates, lysophospholipids and phospholipids. To test whether the plasma metabolite profiles could discriminate patients with POAF from those without POAF, we employed unsupervised PCA to distinguish the group separation. The resultant PCA score plot demonstrated a clear clustering separation between the two groups (Fig. 3a).

Then, the volcano plot was constructed to identify the differentiated metabolic variables responsible for group separation. Altogether, a total of 31 metabolic alterations, including 17 annotated metabolites and 14 unknown ion variables (Fig. 3b). Based on these differentiated metabolic alterations, the calculated PCA score plot could remarkably differentiate POAF and non-POAF subjects (Fig. 3c). The expression levels of the 17 annotated metabolites were plotted as heat map (Fig. 3d). Notably, the resultant graph indicated that these altered metabolites were dominated by a variety of gut microbiota metabolism-related bile acid (BA) species. Patients with POAF had higher levels of three primary BAs (glycochenodeoxycholic acid, taurochenodeoxycholic acid, and cholic acid) and six secondary BAs (7−ketolithocholic acid, hyocholic acid, deoxycholic acid, taurodeoxycholic acid, lithocholic acid, and ursodeoxycholic acid) than patients without POAF. Furthermore, we also observed patients with POAF exhibited increased levels of TMAO and decreased levels of arginine, methionine sulfoxide, benzoic acid, guanosine, inosine, indoleacrylic acid than non-POAF subjects.

### Plasma levels of short-chain fatty acids are increased in patients with POAF

**Fig. 4:**
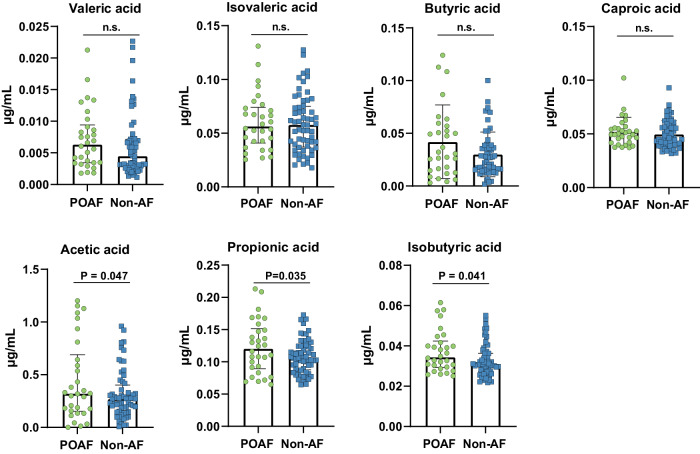
Plasma levels of seven short-chain fatty acid levels in patients with and without POAF. n.s. no significances in univariate analysis, POAF post-operative atrial fibrillation, non-AF non-atrial fibrillation.

Short-chain fatty acids (SCFAs), the metabolic productions from the fermentation of glucose and non-digestible dietary fiber by gut microbiota, have been as the potential contributors to atrial fibrillation pathogenesis. Therefore, the quantitative SCFAs profiling was also performed to explore whether the SCFA species are associated with POAF in patients undergoing isolated CABG. The plasma concentrations of seven SCFAs, including valeric acid, isovaleric acid, butyric acid, caproic acid, acetic acid, propionic acid, and isobutyric acid of the matching two groups were depicted in Fig. 4. The results demonstrated that patients with POAF had higher plasma levels of acetic acid, propionic acid, and isobutyric acid than patients who survived from POAF events. However, no differences were observed in valeric acid, isovaleric acid, butyric acid, and caproic acid between the two groups.

### POAF-altered metabolites are associated with a variety of gut microbiota and functional pathways

**Fig. 5:**
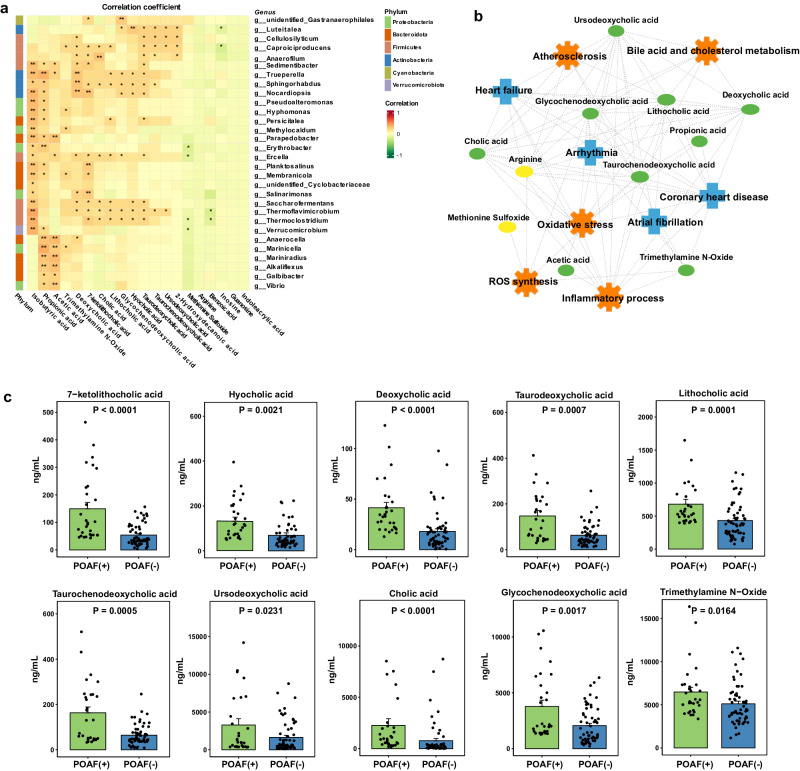
Bio-functional and quantitative analysis of gut microbiota-associated metabolites. **a** The Spearman correlation-based heatmap between differential metabolites and dominant bacteria at phylum and genus levels. *P < 0.05, **<0.01. **b** Ingenuity Pathway Analysis-based functional network between the gut microbial metabolites and enriched biological pathways/diseases. Ellipsoids and nodes represent metabolites and functional pathways/diseases, respectively. Green and yellow ellipsoids represent metabolites that were increased and decreased in the plasma samples of POAF patients compared to non-POAF patients. **c** Dot histogram of the MRM-based quantitative levels of BAs and Trimethylamine N−Oxide in patients with and without POAF. Mann‒Whitney *U* test was used for each comparison.

We subsequently performed Spearman’s rank correlation analysis to assess the correlation of the POAF-associated serum metabolites with microbiome at the phylum and genus levels (Fig. 5a). The results indicated that the three SCFAs, including acetic acid, propionic acid, and isobutyric acid, were positively correlated with Bacteroidota and Proteobacteria. In addition, we observed that most of primary and secondary BA species were significantly associated with Firmicutes and Actinobacteria. To characterize the biological function and latent diseases of POAF-associated metabolites, the functional relationship network was constructed based on the database of Ingenuity Pathway Analysis. The resultant network revealed that those POAF-altered metabolites were significantly related to coronary heart disease, atrial fibrillation, arrhythmia, and heart failure, and were associated with bile BA metabolism, oxidative stress, and inflammation (Fig. 5b). Most strikingly, the accumulation of BA species, TMAO, acetic acid, propionic acid, and the reduction of arginine and methionine sulfoxide in the serum of patients with POAF was closely associated.

### A small panel of secondary bile acids exhibited significant performance in predicting POAF events

In the untargeted metabolite profiles, the most prominent metabolomic characteristics of patients with POAF events were the increased TMAO and nine BA species (Fig. 3d). BAs and TMAO are important metabolic substrates or derivatives in gut microbiota metabolism. In order to verify the qualitative and quantitative accuracy of BAs and TMAO and test whether their real concentrations in the plasma samples were statistically changed between patients with and without POAF, MRM-based targeted analysis of these altered metabolites was performed. As shown in Fig. 5c, all of these metabolites in absolutely quantitative analysis were also statistically different between patients with and without POAF (all *P* values < 0.05).

**Fig. 6:**
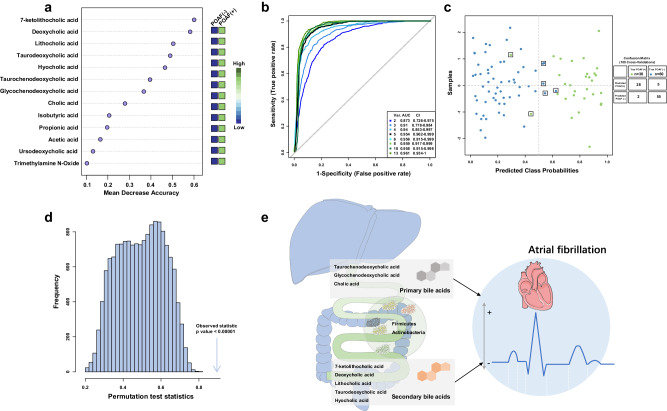
Gut microbiota-derived secondary BAs are significantly associated with POAF. **a** The important POAF-associated metabolites are ranked by using the values of mean decrease accuracy in Random Forest analysis. **b** ROC curves generated from MCCV-based multivariate Random Forest models with different numbers of top important metabolites. The AUC value under the ROC curves and its 95% CI are shown. **c**, **d** Posterior classification probability plot (100 cross-validation) and the permutation test plot (1000 times) based on the multivariable Random Forest model using the top five secondary BAs. **e** Schematic mechanisms underlying the roles of the dominant bacteria in regulating the secondary BAs metabolism.

Then, we collected the differentiated gut microbiota-associated metabolites (nine BAs, three SCFAs, and TMAO) in targeted metabolite profiling for further POAF-related metabolite marker selection and risk model construction. Random forest (RF), an ensemble supervised learning method for variable reduction and selection, was employed to calculate the variable importance and select the optimal marker panel in predicting POAF. The variable importance of each metabolic measure was ranked by using the values of mean decrease accuracy and summarized in Fig. 6a. The results demonstrated that the top eight metabolites were consisted of five secondary BAs and three primary BAs.

Based on the different numbers of top important metabolic variables in mean decrease accuracy plot (from 2 to 13), the Monte Carlo cross validation analysis of multivariable RF models were performed to select the optimal marker panel that can maintain a maximized performance in predicting POAF events. As shown in Fig. 6b, the combination of the top five metabolites (including 7-ketolithocholic acid, deoxycholic acid, lithocholic acid, taurodeoxycholic acid, and hyocholic acid) showed a significant performance in predicting POAF (AUC-ROC value = 0.954), while the additional features had little effect on the values of AUC-ROC. Furthermore, the posterior classification probability plot (Fig. 6c) of these five secondary BAs demonstrated that correct POAF prediction rate was 93.3% (28 in 30). The reliability of the established RF model was also confirmed by the permutations plot (*P* values < 0.00001; Fig. 6d). These results indicated that the secondary BAs produced by the gut microbiota (Fig. 6e) might play a key role in the development of POAF.

### Evaluation of the marker panel in predicting POAF events in an independent validation cohort

In the discovery cohort, the combination of five gut microbiota-derived secondary BAs showed powerful performance in predicting POAF events. To test the predictive performance of this selected marker panel, the targeted analysis of these five secondary BAs and RF model evaluation were performed in an independent validation cohort. A total of 367 patients undergoing isolated CABG constituted the validation cohort. 114 patients developed an POAF event in post-operation 1–6 days. As shown in Supplementary Table 2, no statistical differences were observed in the demographics and clinical characteristics of patients with and without POAF in the validation cohort.

**Fig. 7:**
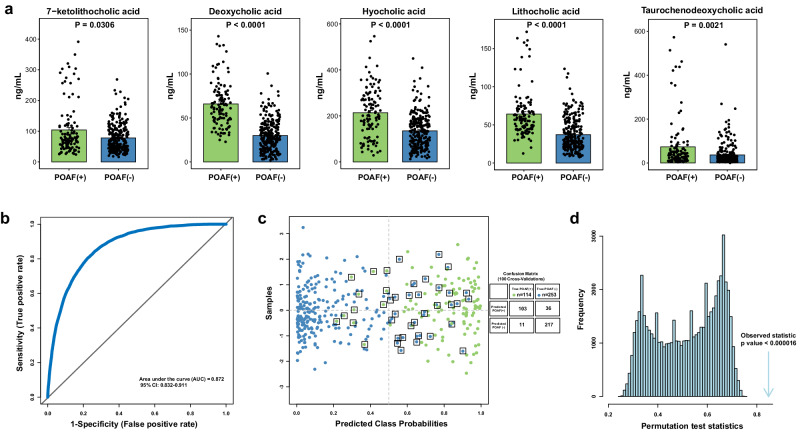
Quantitative analysis and model validation of secondary BAs in validation cohort. **a** Dot histogram of the plasma levels of five secondary BAs in the patients with and without POAF from the validation cohort. Mann‒Whitney *U* test was used for each comparison. **b** Random Forest-based ROC curves using the five secondary BAs. The AUC value under the ROC curves and its 95% CI are shown. **c** Posterior classification probability plot based on 100 cross-validation. **d** The permutation test plot (1000 times).

Notably, in the targeted analysis of five secondary BAs (Fig. 7a), the plasma levels of 7-ketolithocholic acid, deoxycholic acid, lithocholic acid, taurodeoxycholic acid, and hyocholic acid were also identified to be differentially expressed at baseline (all *P* values < 0.05) between subjects who developed POAF events and patients without events. RF model using the selected five secondary BAs was highlighted with a significant prediction performance (Fig. 7b; AUC-ROC value = 0.872). In the posterior classification probability analysis (Fig. 7c), 103 patients (90.4%, 103 in 114) were correctly predicted with an occurrence of POAF event. The predictive accuracy of five secondary BAs-based RF model in validation cohort was also validated by the 1000-times random permutation test with a significant *P* value < 0.000016 (Fig. 7d).

## Discussion

The gut microbiota and its metabolites have been proposed as cofactors in the progression and development of AF through their interaction with multiple functional pathways via the gut–heart axis. In this study, for the first time, we employed 16S rRNA sequencing and targeted/untargeted metabolomic profiling to identify the prospective alterations in the fecal microbiome and plasma metabolome that discriminate POAF from non-POAF in patients undergoing isolated CABG. We demonstrated that POAF was associated with a variety of gut microbial alterations, mainly including bacteria Actinobacteria and Firmicutes and specific SCFAs and BAs produced by gut microbiome. Furthermore, we found that the perturbed gut microbiota-dependent metabolites were significantly enriched in atrial fibrillation, secondary bile acid metabolism, and inflammation processes. Finally, the RF model demonstrated the combination of five gut microbiota-derived secondary BAs provided high accuracy in determining POAF event in two prospective cohorts, suggesting the prognostic potential of microbial markers for the prediction and intervention of POAF.

Epidemiological studies have identified several major risk factors for the development of POAF, but large-scale studies have shown that preoperative, intraoperative, and postoperative factors can only explain a small portion of the variation in POAF risk^2^. The gut microbiota was considered as a metabolic organ that not only contributes to nutrient absorption and energy production but also generates various metabolites (such as BAs, SCFAs, and indole derivatives) that can be distributed throughout the host via the circulation, affecting multiple biological processes^24^. Recent studies have strongly supported the role of microorganisms in POAF and cardiovascular disease through the interaction between metabolites and the host^11,25^.

In this study, our results revealed significant differences in microbiota abundance and diversity in the gut microbiota structure in patients who developed POAF compared to those survived POAF. At phylum-level, we found that two gut microbiota Actinobacteria and Firmicutes were significantly increased in POAF patients compared to non-POAF patients. Firmicutes (also known as Bacillota) and Actinobacteria (also known as Actinomycetota) have been reported to be the major bacteria involving the secondary BA metabolism via convert primary BAs to the major secondary BAs. BAs can be categorized as free or conjugated, as well as primary or secondary based on their source. Interestingly, the correlation analysis also indicated that the plasma concentrations of primary BAs (including cholic acid, taurochenodeoxycholic acid, and glycochenodeoxycholic acid) and secondary BAs (e.g., hyocholic acid, taurodeoxycholic acid, deoxycholic acid) were significantly increased in POAF and were positively related to Actinobacteria and Firmicutes. These findings demonstrated a remarkable perturbed gut microbiota-bile acid axis in POAF.

Primary BAs as the essential components of bile, are directly synthesized in the liver from cholesterol, while secondary BAs are produced by intestinal bacteria through the 7α dehydroxylation of primary BAs. Dysbiosis of gut microbiota can lead to abnormal BA metabolism, resulting in pathologically elevated BAs that can affect cardiac electrical activity and induce arrhythmias through various mechanisms^26–28^. Studies have shown that pro-bile acids like chenodeoxycholic acid can induce cardiac fibrosis through inflammation^29^. BAs rely on muscarinic M2 receptors to induce arrhythmias^30^. Additionally, Gorelik et al.^31^ showed that BAs stimulate the release of calcium ions, which may cause arrhythmias through calcium-activated currents or the Na + /Ca^2+^ exchanger (NCX). Moreshwar et al.^32^ found that excess BAs can also impair fatty acid oxidation in cardiomyocytes, leading to cardiac dysfunction known as cholestatic heart disease.

In most cases, the clinical used POAF-related biomarkers, such as N-terminal pro-B-type natriuretic peptide, C-reactive protein, high-sensitivity cardiac troponin T, interleukin 6, cannot accurately predict POAF after cardiac surgery^33,34^. Therefore, the identification of effective biomarkers to monitor susceptibility is crucial for preventing POAF. In this study, we used the quantitative metabolomics and multivariable RF model to explore whether the gut microbiota-dependent metabolites could accurately differentiate POAF and non-POAF in patients undergoing isolated CABG surgery. Our results indicated that the combination of five secondary BAs exhibited remarkable performances in predicting POAF events in the matched discovery cohort set as well as the independent larger validation cohort set. These findings indicated that gut microbiota and its associated secondary BAs hold promise as diagnostic biomarkers or therapeutic targets. Further research is needed to investigate the mechanisms of action of the gut–bile acids axis in the progression and development of POAF.

Another important finding of our study was that the plasma concentrations of three SCFAs including acetic acid, propionic acid, and isobutyric acid were significantly higher in patients with POAF compared to patients without POAF. SCFAs are produced by the gut microbiota through the metabolism of resistant starch, polysaccharides, and proteins in dietary fiber^19^. However, SCFAs differ in their harmful and beneficial properties. Previous studies found that acetate could affect blood pressure and lower heart rate and had a negative inotropic effect on cardiac contractility^35^. Additionally, acetate might contribute to dyslipidemia and promote obesity by enhancing the secretion of insulin and ghrelin^36,37^. Propionate might stimulate neuronal firing and norepinephrine (NE) synthesis and release and promote inflammation via free fatty acid receptor (FFAR)-3^38^. Furthermore, propionate could also reduce susceptibility to ventricular tachycardia by affecting connexin 43 in cardiomyocytes^39^. Thus, the increased plasma levels of acetate and propionate might play a role in the development of POAF through modifying the atrial fibrillation risk factors and inflammation processes.

Our results also identified that several inflammation and oxidative stress-related metabolites were differentially expressed between POAF and non-POAF patients. Trimethylamine N-oxide (TMAO), a well-studied harmful microbial metabolite, has been suggested to play a role in the pathogenesis of cardiovascular disease Possible mechanisms mainly included inflammation, oxidative stress, and DNA damage^40^. Notably, our results revealed a significant elevation of TMAO in the plasma samples from patients with POAF compared to patients without POAF. Previous evidence incited that raised TMAO might enhance the infiltration of M1 macrophages in atria and increase the expression of Casp1-p20 and cleaved-GSDMD^41^, leading to atrial structural remodeling and increasing susceptibility to POAF. In addition, we also observed two antioxidant and anti-inflammatory metabolites, arginine and methionine sulfoxide were significantly decreased in the plasma samples of POAF patients. These findings were in according to our recent study on investigating the POAF-associated metabolite alterations in the pericardial fluid and serum samples using the targeted metabolite profiling^11^.

Our study has several limitations. First, 16S amplicon sequencing provided limited taxonomy for functional analysis, and future studies should consider performing microbial metagenome analysis. Second, since POAF manifests as a distant postoperative relapse, future studies should explore changes in flora and metabolites in patients with distant POAF relapse. Clinical intervention studies involving dietary manipulation (including probiotics) and fecal transplantation in POAF patients are needed to determine whether the gut microbiota and microbial-derived metabolites can be modified as risk factors for the development and progression of POAF. Finally, although our sample size was larger than previous reports, the two-cohort set of patients was collected from single center. A larger and multi-center studies are needed to validate our main findings.

In summary, the present study demonstrated profound alterations in gut microbiota and its metabolites were associated with POAF. The combination of specific secondary BAs had significant potential for predicting POAF events after CABG procedure. These findings revealed a potential role of gut-heart axis in the causes of POAF, providing potential therapeutic target and intervention strategy after cardiac surgery.

## Methods

### Ethics statement

The study protocol was approved by the Medicine Ethics Committee of Beijing An-Zhen Hospital (Beijing, China) and adhered to the Declaration of Helsinki (Approval no. 2022128X). Written informed consent was obtained from all participants.

### Subjects and study design

A total of 184 patients in Beijing Anzhen Hospital were included as the discovery cohort between January 2022 and December 2022. Another 425 patients in Beijing Anzhen Hospital were included in the validation cohort between January 2023 and January 2023. Inclusion criteria were: participants who received isolated CABG in Beijing Anzhen Hospital. Exclusion criteria for the study were: (1) patients with gastrointestinal diseases; (2) patients with long-term antibiotic therapy or probiotics; (3) patients with preoperative arrhythmia. After screening, 158 individuals were enrolled in the discovery cohort, and 367 patients were enrolled in the validation cohort. All patients in the research signed informed consent before sample collection. The study was approved by the Ethics Committee of Beijing Anzhen Hospital and was conducted following the Declaration of Helsinki.

POAF is defined as a new onset of AF after cardiac surgery sustained for 30 seconds or more^42^ Patients were placed on continuous 24-h cardiac monitoring after surgery until patients were discharged from the hospital and 12-lead ECGs were obtained to confirm rhythm abnormalities. The occurrence of the first documented POAF episode was the study end point. After surgery, there were 50 patients with POAF and 108 patients without POAF in the discovery cohort and 114 patients with POAF and 253 patients without POAF in the validation cohort. Patients matched based on the propensity score matching (PSM) by the nearest neighbor matching algorithm, and the optimal parameters (caliper and ratio) were determined through the covariate balance analysis using the standardized mean difference. “Caliper” defines the maximum distance of the propensity score between 2 samples, and “ratio” defines how many control samples could be matched to each disease sample. Finally, the optimal parameters (caliper = 0.25 and ratio = 2) of the 1:2 PSM were determined through the covariate balance analysis. According to the above matching process, a total of 90 patients were obtained, including 30 POAF (+) and 60 POAF (−) in the discovery cohort by matching the clinical variables with statistical differences and variables with non- statistical difference but may have a potential impact, including ages, sex, hospital length of stay, LVEF, number of grafts, and TSH.

### Fecal microbiome and plasma metabolome profiling

After signing the informed consent, we requested that the participants follow a uniform diet for 3 consecutive days and maintain an overnight fasting state of 10–12 h before blood and fecal sample collection. The blood sample was collected from the antecubital vein of patients. All samples were stored at −80 °C until further analysis. Detailed methods of 16S rRNA gene sequencing, and untargeted/targeted metabolomics analysis are available in the Supporting Information.

### Statistical analysis

Continuous variables with a normal distribution are presented as the mean ± standard deviation (SD); otherwise, abnormally distributed data are presented as the median (interquartile range). Student’s *t* test and Mann–Whitney *U* test were used for the comparison of normally distributed data and non-normally distributed data, respectively. Categorical variables were summarized by frequency (N) and percentages (%) and compared using Chi-square test. *P* < 0.05 was considered significant. All analyses were performed by SPSS Statistics Version 25 (IBM Corp., Armonk, NY, USA).

The raw untargeted metabolomic data were transformed to Progenesis QI (Waters, Manchester, U.K.) for deconvolution, alignment, retention time correction, and metabolite identification (based on the QI MetaScope database, METLIN database, HMDB database, LIPIDMAPS database, and in-house metabolite library). The normalized metabolite features that were absent in more than 10% of pooled quality control (QC) injections throughout analysis were removed. From the remaining features, those with more than 20% relative standard deviation (RSD) in peak intensity across pooled QC injections were also removed. The multivariate statistical analysis for the metabolomic data matrix was performed using SIMCA-P software (v14.1, Umetric, Umeå, Sweden). Unsupervised principal component analysis (PCA) was applied to gain a comprehensive view of the sample distribution, group separation, and assess the outlier samples using the pareto-calculation of principal components (PCs). The optimized number of principal component (PC) was determined by R2 and Q2 values as follows: more than 50% of original variables have been explained by the selected PCs (cumulative R2 values > 0.5), and the cumulative Q2 values of the selected PCs were decreased after adding a new PC. Differentiated metabolites were selected by using a volcano plot (log2-fold change vs. -Log10 FDR-adjusted P value), and a fold change value > 2.0 or 0.5 and an adjusted P value < 0.05 represent the significant importance of the metabolic variables in differentiating POAF from non-POAF. The latent relationship network between POAF-associated metabolites and functional pathways/diseases was generated based on Function Analysis, Connect Analysis, and Path Explorer by using Ingenuity Pathway Analysis (IPA, QIAGEN Inc., German).

Spearman’s rank coefficients were used to investigate the correlation between differentiated metabolites and alterations at the genus and phylum levels. The random forest (RF) algorithm was used to estimate the association between differentiated metabolites and POAF events and select the important biomarker panel using R software (https://cran.r-project.org/index.html). The function Random Over-Sampling Examples (ROSE) from the R package ‘ROSE package’ was applied to reduce the data imbalance rate. The ntree value and mtry value were set at 500 and 5, respectively. The other hyperparameters were set at default settings of the R package ‘Random Forest’. The variable importance was evaluated by using the values of mean decrease accuracy. Monte Carlo cross-validation (MCCV) was used to generate a multivariate RF model for selecting the optimal metabolite marker panel. The performance of multivariate RF models was assessed using the values of area under the receiver-operating characteristic curve (AUC-ROC). The reliability of the optimal RF model was evaluated by the number of misclassifications in the posterior classification probability plot (100 cross-validation) and permutation test (*n* = 1000 times).

## Supplementary information

Supporting Information

## Data Availability

Data are available upon reasonable request.

## Code availability

The data in this study are available from the corresponding author for academic purpose-request.
