# Supplementary material for: Gut microbiota metabolism disturbance is associated with postoperative atrial fibrillation after coronary artery bypass grafting
Source: NPJ Cardiovasc Health. 2024 Jun 3;1:5. doi: 10.1038/s44325-024-00003-z (PMC12912376; doi:10.1038/s44325-024-00003-z)
Supplement: Supplementary file 1 — Supporting Information [file 44325_2024_3_MOESM1_ESM.pdf]

Supporting Information

Gut microbiota metabolism disturbance is associated with postoperative atrial fibrillation after coronary artery bypass grafting

Yuhua Liu[1*], Zhiyong Du[1*#], Yingyuan Lu[2*], Ying Ma[3], Yunxiao Yang[1], Florian Osmanaj[1], Yifan Zhang[2], Xiaoyu Guo[2], Yanwen Qin[1], Xiubin Yang[1#], Kun Hua[1#]

[1] Beijing Institute of Heart Lung and Blood Vessel Disease, Beijing Anzhen Hospital, Capital Medical University, Beijing China

[2] State Key Laboratory of Natural and Biomimetic Drugs, School of Pharmaceutical Sciences, Peking University, Beijing, China

[3] State Key Laboratory for Quality Ensurance and Sustainable Use of Dao-di Herbs， National Resource Center for Chinese Materia Medica, China Academy of Chinese Medical Sciences, Beijing, China

[*] Theses authors contributed equally: Yuhua Liu, Zhiyong Du, Yingyuan Lu.

[#]Correspondence authors: Zhiyong Du, Xiubin Yang, Kun Hua. email: duzhiyong1989@163.com; xiubinyang@hotmail.com; kunhua@mail.ccmu.edu.cn

SUPPLEMENTARY METHODS

16S rRNA gene sequencing and data analysis

Fecal samples (about weight 5 g) were collected from participants before surgery, suspended in fecal storage solution and then snap frozen and stored at −80 °C. Bacterial DNA was extracted using the CTAB method. DNA concentration and purity were monitored on 1% agarose gels. According to the concentration, DNA was diluted to 1 ng/L. 16S rRNA gene sequencing was performed by Metware Ltd. (http://www.metware.cn/). Amplicon generation and quantification and qualification of PCR products were performed according to standard protocols. Sequencing libraries were generated using TruSeq® DNA PCR-Free Sample Preparation Kit (Illumina, USA) following the manufacturer's recommendations, and index codes were added. The library quality was assessed on the Qubit@ 2.0 Fluorometer (Thermo Scientific, USA) and Agilent Bioanalyzer 2100 system.

Sequences analysis were performed by Uparse software (Uparse v7.0.1001, http://drive5.com/uparse/) (Edgar 2013). Sequences with 97% similarity were assigned to the same OTUs. A representative sequence for each OTU was screened for further annotation. OTUs abundance information were normalized using a standard sequence number corresponding to the sample with the least sequences. Alpha diversity analysis was applied to identify community richness and diversity, including Chao1 and Shannon indices. Principal Coordinate Analysis (PCoA) was performed to provide principal coordinates and visualize the classification of samples.

Untargeted plasma metabolomics analysis

A total of 50 μL of each sample after thawed was mixed with 250 μL of extraction solution (acetonitrile: methanol = 1:4) containing internal standards. The mixture was vortexed for 5 min and then centrifuged at 12,000 rpm for 10 min at 4 °C. Subsequently, 250 μL of supernatant was taken into a new centrifuge tube and placed in a -20 °C refrigerator for 30 min. The supernatant was centrifuged at 12,000 rpm for 10 min, and 180 μL of the supernatant was transferred for LC–MS analysis.

Metabolomic profiling was performed on a high-resolution UPLC-SYNAPT Xevo-G2 XS Q-TOF/MS system (Waters Corporation, Milford, USA). The separation of metabolites was performed on an ACQUITY UPLC HSS T3 C18 column (100 × 2.1 mm, 1.8 μm; Waters Corp., Milford, MA, USA). The column temperature was maintained at 40 °C, and the flow rate remained constant at 0.4 mL/min. The sample injection volume of each sample was 2 μL. The column was eluted with 5% mobile phase B (0.1% formic acid in acetonitrile) at 0 minute followed by a linear gradient to 90% mobile phase B (0.1% formic acid in acetonitrile) over 11 minutes, held for 1 minute, and then returned to 5% mobile phase B within 0.1 minute, held for 1.9 minutes. The MS detection parameters were as follows: the capillary voltage was 3.0 kV for positive ion mode and 2.3 kV for negative ion mode; the sampling cone voltage was 35 V; the cone gas rate was set at 50 L/h; and the desolvation gas temperature and desolvation gas flow were 450 °C and 700 L/h, respectively. The source temperature was set at 110 °C. All analyses were acquired using a LockSpray interface to ensure accuracy and reproducibility. Data were collected in centroid mode from 50 to 1100 Da. The collision energy parameters ranged from 5 eV to 65 eV.

GC–MS-based quantitative analysis of SCFAs

Plasma concentrations of SCFAs were detected by MetWare (http://www.metware.cn/) based on the Agilent 7890 gas chromatograph (GC)-7000D mass spectrometer (MS) platform. Briefly, a total of 50 μL of plasma sample after thawing was mixed with 200 μL of phosphoric acid (0.5% v/v) solution. Then, the mixture was vortexed for 10 min, ultrasonicated for 5 min, and centrifuged at 12000 r/min for 10 min at 4 °C. One hundred microliters of supernatant was moved into a 1.5 mL centrifugal tube with 250 μL methyl tert-butyl ether (MTBE) containing internal standards. The mixture was vortexed for 3 min and ultrasonicated for 5 min, followed by centrifugation at 12000 r/min for 10 min at 4 °C. The supernatant was collected and used for GC–MS analysis. Detection of SCFAs was performed using a DB-FFAP column (30 m length × 0.25 mm i.d. × 0.25 μm film thickness, J&W Scientific, USA). Injection was made in split mode with a split ratio of 5:1, and the injection volume was 1 μL. Helium was used as the carrier gas at a flow rate of 1.2 mL/min. The oven temperature was held at 50 °C for 1 min, raised to 220 °C at a rate of 18 °C/min and held for 5 min. The injector inlet and transfer line temperatures were 250 °C and 230 °C, respectively. The multiple reaction monitoring (MRM) transitions were as follows: valeric acid (60→42; RT, 5.287 min); isovaleric acid (60→42; RT, 4.875 min); butyric acid (60→42; RT, 4.614 min); caproic acid (73→55; RT, 5.872 min); acetic acid (60→43; RT, 3.389 min); propionic acid (74→28.1; RT, 4.008 min); and isobutyric acid (73→55; RT, 4.002 min).

LC–MS-Based targeted and quantitative analysis of BAs and trimethylamine N−Oxide

The plasma levels of nine BAs and trimethylethylamine N−oxide (TMAO) were determined by MetWare (http://www.metware.cn/) based on the AB Sciex triple quadrupole-linear ion trap mass spectrometer (QTRAP) 6500 LC–MS/MS platform. Metabolite extraction and separation were performed using the same approach used for untargeted metabolomics. The column temperature was maintained at 40 °C, and the flow rate remained constant at 0.4 mL/min. The sample injection volume of each sample was 2 μL. The MS/MS parameters were as follows: ion spray needle voltage, 5500 V/− 4500 V; GS1, GS2, and CUR were set as 45 psi, 45 psi, and 35 psi, respectively; turbo gas temperature, 550 °C; collisional activated dissociation (CAD) gas, medium level. Delustering potential (DP) and collision energy (CE) for individual MRM transitions were determined with further DP and CE optimization. A specific set of MRM transitions was monitored for each metabolite as follows: TMAO (75.8→58.7; DP: 20; CE: 14); 7−ketolithocholic acid (389.5→215.1; DP: 50; CE: 20); hyocholic acid (407.5→407.5; DP: -150; CE: -10); deoxycholic acid (391.2→391.2; DP: -168; CE: -12); taurodeoxycholic acid (498.2→79.9; DP: -122; CE: -126); lithocholic acid (375.2→375.2; DP: -196; CE: -11); taurochenodeoxycholic acid (498.2→124; DP: -166; CE: -12); ursodeoxycholic acid (391.2→391.2; DP: -178; CE: -10); cholic acid (407.2→407.2; DP: -196; CE: -16); and glycochenodeoxycholic acid (448.3→448.3; DP: -100; CE: -20).

Supplementary Table 1. Demographic and clinical characteristics of participants in discovery cohort.

Variable	Unmatched			1:2 Propensity score matched		
	**POAF (+) (N = 50)**	**POAF (-) (N = 108)**	**P-value**	**POAF (+) (N = 30)**	**POAF (-) (N = 60)**	**P-value**
**Pre-operative**						
Age (years)	63.52 ± 4.66	62.23± 5.89	0.175	63.66 ± 5.10	62.47± 7.77	0.448
Male sex	33 (66.00)	59 (54.63)	**0.055**	22 (73.33)	43 (71.67)	0.088
BMI (kg/m^2)	26.89 ± 3.19	26.52 ± 1.89	0.365	26.37 ± 3.43	26.22 ± 2.32	0.807
Hypertension	26 (52.00)	57 (52.78)	0.973	17 (56.67)	35 (58.33)	0.880
Diabetes mellitus	21 (42.00)	68 (62.96)	0.895	14 (46.67)	27 (45.00)	0.881
COPD	3 (6.00)	11 (10.19)	0.743	2 (6.67)	3 (5.00)	0.784
Previous PCI	14 (28.00)	23 (21.30)	0.469	8 (26.67)	14 (23.33)	0.730
History of Stroke	7 (14.00)	9 (8.33)	0.284	4 (13.33)	7 (11.67)	0.821
Previous MI	11 (22.00)	27 (25.00)	0.702	5 (16.67)	11 (18.33)	0.845
Current Smoker	35 (70.00)	63 (58.33)	0.219	19 (63.33)	37 (61.66)	0.878
Drinking history	19 (38.00)	34 (31.48)	0.422	8 (26.67)	13 (21.67)	0.600
BNP (pg/mL)	235.65 (80.50, 455.35)	265.41 (60.72, 487.55)	0.319	247.72 (85.50, 453.25)	263.37 (61.92, 477.35)	0.439
HbA1c (%)	6.32 ± 3.35	6.10 ± 0.99	0.531	6.25 ± 3.41	6.12 ± 1.85	0.815
CREA (mmol/L)	65.74 ± 14.52	69.10 ± 15.14	0.191	66.88 ± 15.32	68.50 ± 17.28	0.664
TG (mmol/L)	1.70 (1.01, 1.99)	1.60 (1.04, 1.99)	0.152	1.68 (1.04, 1.92)	1.62 (1.07, 1.94)	0.764
TC (mmol/L)	4.03 ± 0.46	4.15 ± 0.71	0.276	4.05 ± 0.78	4.20 ± 0.70	0.359
HDL-C (mmol/L)	1.08 ± 0.35	1.15 ± 0.17	0.092	1.09 ± 0.21	1.13 ± 0.20	0.381
LDL-C (mmol/L)	2.20 ± 0.54	2.08 ± 0.67	0.269	2.16 ± 0.66	2.11 ± 0.54	0.720
TSH (mIU/L)	1.70 (1.02, 3.24)	1.93 (1.01, 3.95)	0.083	1.79 (1.12, 3.01)	1.89 (1.07, 3.76)	0.398

fT_3 (pmol/L)	4.48 ± 0.25	4.55 ± 0.35	0.206	4.49 ± 0.38	4.59 ± 0.55	0.374
fT_4 (pmol/L)	11.83 ± 1.20	11.48 ± 1.42	0.133	11.73 ± 1.39	11.52 ± 1.82	0.580
LVEF (%)	59.98 ± 4.19	61.28 ± 3.91	**0.059**	60.60 ± 3.45	61.20 ± 5.17	0.567
LAD (mm)	36.55 ± 4.09	35.25 ± 5.30	0.127	36.22 ± 4.31	35.35 ± 3.55	0.311
**Intra-operative**						
Duration of surgery. (hours)	4.27 ± 0.50	4.19 ± 0.85	0.538	4.21 ± 0.56	4.20 ± 0.83	0.953
Number of grafts	3.40 ± 0.52	3.59 ± 0.59	**0.053**	3.42 ± 0.80	3.47 ± 0.63	0.747
RBC Transfusion (U)	29 (58.00)	70 (64.81)	0.412	14 (46.67)	35 (58.33)	0.449
**Post-operative**						
Ventilation >24 hours	28 (56.00)	65 (60.19)	0.620	11 (36.67)	27 (45.00)	0.074
Intensive care unit time (hours)	50.00 (20.00, 90.00)	52.00 (24.00, 86.00)	0.475	48.00 (24.00, 86.00)	48 (24.00, 74.00)	0.260
Hospital length of stay (days)	8.00 (7.00, 9.00)	7.00 (7.00, 8.00)	**0.036**	7.00 (6.00, 8.00)	7.00 (6.00, 8.00)	0.967

Data displayed as mean ± standard deviation, median values (interquartile range), and n (%). Differences between groups were analyzed by the Fisher test, Student t test, $\chi 2$ test, or Kruskal-Wallis test. POAF, postoperative atrial fibrillation; BMI, body mass index; COPD, chronic obstructive pulmonary disease; PCI, percutaneous coronary intervention; MI, myocardial infarction; BNP, brain natriuretic peptide; HbA1c, hemoglobin A1C; TG, triglyceride; TC, total cholesterol; HDL-C, high-density lipoprotein cholesterol; LDL-C, low-density lipoprotein cholesterol; TSH: thyroid stimulating hormone; fT3: free triiodothyronine; fT4: free thyroxine; LVEF, left ventricular ejection fraction; LAD, left atrium diameter.

Supplementary Table 2. Demographic and clinical characteristics of participants in validation cohort.

Variable	**POAF (N =114)**	**non-POAF (N = 253)**	**P-value**
**Pre-operative**			
Age (years)	63. 66 ± 2.51	63.79± 2.51	0.600
Male sex	67 (59.29)	166 (65.35)	0.267
BMI (kg/m^2)	26.93 ± 4.55	26.60 ± 3.89	0.477
Hypertension	51 (45.13)	120 (47.24)	0.708
Diabetes mellitus	39 (34.51)	113 (44.49)	0.072
COPD	12 (8.85)	15 (5.91)	0.121
Previous PCI	21 (18.58)	30 (11.81)	0.090
History of Stroke	19 (17.12)	35 (13.78)	0.414
Previous MI	31 (27.43)	27 (21.65)	0.232
Current Smoker	62 (54.87)	119 (46.85)	0.156
Drinking history	29 (25.66)	53 (20.86)	0.313
BNP (pg/mL)	221.65 (75.63, 434.15)	235.41 (55.76, 437.55)	0.483
HbA1c (%)	6.25 ± 5.47	6.30 ± 6.66	0.944
CREA (mmol/L)	63.85 ± 9.89	65.90 ± 10.32	0.076
TG (mmol/L)	1.57 (1.03, 2.09)	1.65 (1.10, 1.93)	0.322
TC (mmol/L)	4.11 ± 0.91	4.19 ± 0.85	0.416
HDL-C (mmol/L)	1.11 ± 0.95	1.15 ± 0.79	0.675
LDL-C (mmol/L)	2.15 ± 0.93	2.04 ± 0.99	0.318
TSH (mIU/L)	1.51 (1.12, 3.94)	1.83 (1.07, 3.73)	0.073
fT_3 (pmol/L)	4.43 ± 0.79	4.59 ± 0.99	0.130
fT_4 (pmol/L)	11.23 ± 1.59	11.10 ± 1.89	0.524
LVEF (%)	58.50 ± 6.20	59.33 ± 5.59	0.205
LAD (mm)	35.32 ± 5.57	35.50 ± 6.63	0.801
**Intra-operative**			
Duration of surgery (hours)	4.53 ± 1.32	4.43 ± 1.55	0.551
Number of grafts	3.35 ± 0.50	3.40 ± 0.73	0.508
RBC Transfusion (U)	71 (62.83)	155 (61.02)	0.742
**Post-operative**			
Ventilation >24 hours	50 (44.25)	109 (42.91)	0.812
Intensive care unit time. (hours)	48.00 (18.00, 92.00)	54.00 (20.00, 88.00)	0.355
Hospital length of stay (days)	7.00 (6.00, 8.00)	7.00 (6.00, 8.00)	0.698

Data displayed as mean ± standard deviation, median values (interquartile range), and n (%). Differences between groups were analyzed by the Fisher test, Student t test, χ2 test, or Kruskal-Wallis test. POAF, postoperative atrial fibrillation; BMI, body mass index; COPD, chronic obstructive pulmonary disease; PCI, percutaneous coronary intervention; MI, myocardial infarction; BNP, brain natriuretic peptide; HbA1c, hemoglobin A1C; TG, triglyceride; TC, total cholesterol; HDL-C, high-density lipoprotein cholesterol; LDL-

C, low-density lipoprotein cholesterol; TSH: thyroid stimulating hormone; fT3: free triiodothyronine; fT4: free thyroxine; LVEF, left ventricular ejection fraction; LAD, left atrium diameter.
